# VDAC1 in the diseased myocardium and the effect of VDAC1-interacting compound on atrial fibrosis induced by hyperaldosteronism

**DOI:** 10.1038/s41598-020-79056-w

**Published:** 2020-12-16

**Authors:** Hadar Klapper-Goldstein, Ankit Verma, Sigal Elyagon, Roni Gillis, Michael Murninkas, Srinivas Pittala, Avijit Paul, Varda Shoshan-Barmatz, Yoram Etzion

**Affiliations:** 1 Cardiac Arrhythmia Research Laboratory, Department of Physiology and Cell Biology, Faculty of Health Sciences, Ben-Gurion University of the Negev, P.O. Box 653, 84105 Beer Sheva, Israel; 2 Regenerative Medicine and Stem Cell Research Center, Ben-Gurion University of the Negev, Beer Sheva, Israel; 3 Department of Life Sciences, Ben-Gurion University of the Negev, Beer Sheva, Israel; 4 The National Institute for Biotechnology in the Negev, Ben-Gurion University of the Negev, Beer Sheva, Israel; 5 Department of Life Sciences and the National Institute for Biotechnology in the Negev, Ben-Gurion University of the Negev, Beer Sheva, Israel; 6 Present Address: Special Center for Molecular Medicine, Jawaharlal Nehru University, New Delhi, India

**Keywords:** Cardiovascular biology, Cardiovascular diseases, Animal disease models

## Abstract

The voltage-dependent anion channel 1 (VDAC1) is a key player in mitochondrial function. VDAC1 serves as a gatekeeper mediating the fluxes of ions, nucleotides, and other metabolites across the outer mitochondrial membrane, as well as the release of apoptogenic proteins initiating apoptotic cell death. VBIT-4, a VDAC1 oligomerization inhibitor, was recently shown to prevent mitochondrial dysfunction and apoptosis, as validated in mouse models of lupus and type-2 diabetes. In the present study, we explored the expression of VDAC1 in the diseased myocardium of humans and rats. In addition, we evaluated the effect of VBIT-4 treatment on the atrial structural and electrical remodeling of rats exposed to excessive aldosterone levels. Immunohistochemical analysis of commercially available human cardiac tissues revealed marked overexpression of VDAC1 in post-myocardial infarction patients, as well as in patients with chronic ventricular dilatation\dysfunction. In agreement, rats exposed to myocardial infarction or to excessive aldosterone had a marked increase of VDAC1 in both ventricular and atrial tissues. Immunofluorescence staining indicated a punctuated appearance typical for mitochondrial-localized VDAC1. Finally, VBIT-4 treatment attenuated the atrial fibrotic load of rats exposed to excessive aldosterone without a notable effect on the susceptibility to atrial fibrillation episodes induced by burst pacing. Our results indicate that VDAC1 overexpression is associated with myocardial abnormalities in common pathological settings. Our data also indicate that inhibition of the VDAC1 can reduce excessive fibrosis in the atrial myocardium, a finding which may have important therapeutic implications. The exact mechanism\s of this beneficial effect need further studies.

## Introduction

Mitochondria play crucial roles in the cellular energy metabolism, cellular redox status, osmotic regulation, pH control, calcium homeostasis, and more^1–3^. In addition, mitochondria play an important role in the regulation of programmed cell death (apoptosis) via release of pro-apoptotic agents and/or disruption of cellular energy metabolism, as well as interacting with apoptotic proteins^4,5^. Therefore, mitochondria are the venue for cellular decisions leading to cell life or death.

Mitochondria energy production is crucial for the heart due to its high energy demands. Mitochondrial dysfunction is associated with the development of numerous cardiovascular diseases such as atherosclerosis, myocardial ischemia–reperfusion (I/R) injury, hypertension, diabetes, cardiac hypertrophy, and heart failure. Therefore, targeting mitochondrial dysfunction is a crucial step in the treatment of a variety of cardiac diseases^6,7^.

A key player in mitochondrial function is the voltage-dependent anion channel 1 (VDAC1), located on the outer mitochondrial membrane (OMM). VDAC1 serves as a mitochondrial gatekeeper mediating the fluxes of ions, nucleotides, and other metabolites across the OMM and controlling the metabolic and energy cross-talk between mitochondria and the rest of the cell. VDAC1 has also been recognized as a key protein in mitochondria-mediated apoptosis, regulating the release of apoptogenic proteins, and interacting with anti-apoptotic proteins^8–10^. In addition to VDAC1, the most abundant isoform, two other isoforms, VDAC2, and VDAC3 are expressed in mammalians and share some functional and structural characteristics. Both VDAC1- and VDAC3-deficient mice are viable, while VDAC2 KO is lethal and this protein is considered to have anti-apoptotic properties^11^.

Apoptotic and stress conditions lead to oligomerization of VDAC1 to form a large flexible pore, allowing the passage of folded proteins like cytochrome c (Cyto c) through the OMM, leading to apoptotic cell death^12,13^. Therefore, VDAC1 is considered as an innovative target for controlling dysregulated cell metabolism and apoptosis^13,14^. Mitochondrial dysfunction and VDAC1 overexpression are associated with various pathologies such as Alzheimer's disease (AD), type-2 diabetes (T2D), cancer, lupus, and other autoimmune diseases^8,15–18^. VBIT-4, a compound that prevents VDAC1 oligomerization, was found to protect against mitochondria dysfunction and apoptotic cell death and to restore cell energy and metabolism^18^. Indeed, VBIT-4 treatment recently demonstrated remarkable efficiency in murine models of T2D and lupus-like syndrome^15,17^. However, this compound has not yet been tested in-vivo in the context of cardiac pathologies.

The myocardium is a major oxygen consumer organ, and its metabolic functions are strictly regulated under normal and stress conditions^19,20^. Impaired metabolism is one of the hallmarks of the diseased and failing heart^19,21^. In addition, apoptotic cell death contributes to the permanent loss of cardiomyocytes in various cardiac pathologies including ischemia–reperfusion (I/R) injury, pathological cardiac hypertrophy, and heart failure (HF)^22–24^. In the HF context, activation of the renin-angiotensin II-aldosterone system (RAAS) has a well-documented detrimental role, and blockade of RAAS can significantly reduce morbidity and mortality^25^ although the outcome of HF patients remains extremely poor even in the presence of optimal medical therapy that includes RAAS blockers^26^. RASS activation is also involved in the pathogenesis of atrial fibrillation (AF), the most common cardiac arrhythmia and a major cause of hospitalizations, stroke, and heart failure progression^27^. The final step of RAAS activation is aldosterone (Aldo) production. Accumulating evidence supports the involvement of excessive Aldo in the promotion of atrial apoptosis and fibrosis^28,29^.

The involvement of VDAC1 in the pathogenesis of cardiac abnormalities is far from being elucidated. However, accumulating evidence suggests that VDAC1 might have pivotal roles in various settings. In the context of cardiac I/R, upregulation of VDAC1 expression and phosphorylation have been shown to augment cardiomyocyte damage, and inhibition of these processes was mechanistically linked to the nutritional preconditioning function of resveratrol^30–33^. Oxidative stress damage in H9c2 myoblasts was reported to increase VDAC1 expression levels and its oligomerization^34,35^. In addition, VDAC1 was found to be involved in detrimental Ca^2+^ transfer from the endoplasmic reticulum (ER) to the mitochondria^36^. During chronic cardiac abnormalities, changes in the expression and function of VDAC1 are less clear compared to the acute I/R setup. Upregulated transcriptional levels of a diverse array of genes including VDAC1 were found in the septal tissue of human patients with hypertrophic cardiomyopathy^37^. In the same direction, Mitra et al.^24^ documented prominent upregulation of VDAC1 expressional levels in a rat model of cardiac hypertrophy induced by renal artery ligation. In-vivo treatment with siRNA against VDAC1 could partially inhibit the apoptotic cell death observed in this model. Interestingly, in the same study, upregulation of VDAC1 was not detected ten days post-myocardial infarction (MI)^24^. In contrast to the findings of Mitra et al.^24^, the expressional levels of VDAC1 were downregulated in a cellular model of cardiomyocyte hypertrophy induced by the α1-adrenergic agonist phenylephrine^38^. Moreover, the anti-hypertrophic effects of peroxisome proliferator activated receptor α (PPAR α) activation in this setting could prevent this downregulation of VDAC1. Thus, it is still unclear how the expression of VDAC1 is affected by common cardiac pathologies including pathological hypertrophy and ischemic cardiomyopathy. In addition, to the best of our knowledge, the role played by VDAC1 in the atria has not been studied.

In the present study, by utilizing commercially available healthy and diseased human samples as well as rat myocardial samples, we demonstrate an increase in the expressed levels of VDAC1 in the setting of common cardiac pathologies including in the post-MI setup, chronic LV dilatation and dysfunction, and hyperaldosteronism. In addition, the VDAC1 oligomerization and apoptosis inhibitor VIBIT-4 was used to elucidate, for the first time, the possible detrimental role of the overexpressed VDAC1 in the rat atria exposed to excessive aldosterone levels, protecting against cell death and fibrosis.

## Methods

### Human cardiac tissue microarrays

Analysis of human cardiac tissues was performed on commercially available microarray slides, which focused primarily on patients with cardiac pathologies [Cat.-No.: 401 4101 and Cat.-No.: 401 4102, Provitro AG (Charitéplatz 1,10117 Berlin, Germany)]. Overall, our analysis quantified VDAC1 expression on 39 tissue samples that were clustered into three different groups based on supplemental clinical data: (a) Normal group (n = 7); patients reported to have a normal heart with no known pathology, (b) MI group (n = 16); patients reported to be post MI. (c) Chronic myocardial disease group (n = 16); patients reported to have various cardiac abnormalities, leading to LV dilatation\dysfunction\hypertrophy. The clinical data of the MI samples further defined them as “acute phase”, “granulation phase”, or “scar\chronic phase”. In our study, the first two definitions were clustered as “MI, Short-term”, while the latter cases were clustered as “MI, Long-term”. Demographic and diagnosis details are summarized in Supplemental Tables S1–S3. Technical Information on the microarrays are detailed online on the supplier data sheets (https://www.provitro.de/fileadmin/provitro-data/TMA/4014101.pdfhttps://www.provitro.de/fileadmin/provitro-data/TMA/4014102.pdf).

### Animal studies

The animal studies reported in this paper were carried out as previously described by our group^37^. Briefly, experiments were performed in strict accordance with the Guide for the Care and Use of Laboratory Animals of the National Institute of Health and were approved by the institutional ethics committee of Ben-Gurion University of the Negev, Israel. All studies were carried out on adult male Sprague–Dawley rats (250–350 g) obtained from Envigo Laboratories (Jerusalem, Israel). The animals were kept under standardized conditions throughout the study, according to home office guidelines: 12:12 light/dark cycles at 20–24 °C with 30–70% relative humidity. Animals were free-fed autoclaved rodent chow and had free access to drinking water. The animals were monitored on a daily basis for signs of stress or inappropriate weight loss, according to guidance from the Ben-Gurion University veterinary services [assured by the Office of Laboratory Animal Welfare, USA (OWLA) #A5060-01, and fully accredited by the Association for Assessment and Accreditation of Laboratory Animal Care International (AAALAC)]. At the end of all experiments, animals were euthanized under deep anesthesia.

### Rat cardiac tissue samples

The rat cardiac tissue samples that we used to evaluate VDAC1 expression in the presence of sham, hyperaldosteronism, and MI treatments were all from a previously reported study in which rats were instrumented with an atrial pacing and recording device for AF burden evaluation over a period of 4 weeks^39^. Briefly, sham animals were implanted with osmotic mini-pumps delivering PEG-400 (solvent). Aldosterone (Aldo) rats were implanted with osmotic mini-pumps delivering aldosterone (1.5 µg/h) dissolved in PEG-400, and the MI group included rats subjected to left coronary artery ligation. For additional details, see Klapper-Goldstein et al. 2020^39^.

### VBIT-4 treatment in rats with hyperaldosteronism

Twenty rats co-implanted with atrial pacing and recording devices and osmotic mini-pumps (ALZET Model 2004) delivering aldosterone (1.5 µg/h) subcutaneously recovered for 7 days. Thereafter, the rats were randomly divided to receive in the drinking water VBIT-4 (0.3 mg/ml) or control solution (0.1% DMSO only) for 3 consecutive weeks. The VBIT-4 and control treatment water was replaced on a daily basis. Taking into account an estimated water consumption of 8–10 ml/100 gr per rat, the daily dose of VBIT-4 was about ~ 25–30 mg/Kg. For preparation of the VBIT-4, drinking water was titrated to pH = 4 with HCL. Then VBIT-4 dissolved in pure DMSO was added in a dropwise manner into the acidic water to reach a final concentration (0.3 mg/ml). The control treatment was adjusted to have a similar pH and DMSO concentration (0.1%). Fluid consumption was verified on a daily basis.

### AF burden evaluation following VBIT-4 treatment

The implantable device and electrophysiological (EP) apparatus were previously described in detail by our group^39,40^. Briefly, for device implantation, animals were anesthetized (IM ketamine/xylazine 75/5 mg kg^−1^) and mechanically ventilated. Under sterile conditions, an atrial electrode was implanted on the right atrium, peripheral electrodes were positioned in the animal’s back and a connector was exteriorized through the skin. A shielding ring with four plastic restraints was inserted over the connector, sutured to the skin, and glued to the connector over four plastic restraints to prevent the extraction of the device over time. Post-operative recovery and analgesia were performed as described previously^39^. The animals were placed in the dedicated recording chambers for the AF substrate evaluation, while having free access to food and drinking bottles.

The AF burden evaluation was done two and four weeks following device implantation, as recently described in detail^39^. Briefly, the protocol included 20 consecutive atrial pacing bursts [20S, double diastolic threshold, 10 ms cycle length (CL)] followed by recording of the pacing outcome. Arrhythmic episodes were identified by recordings demonstrating more than one atrial signal per ventricular complex and an atrial signal which was clearly different in morphology in comparison with the regular atrial signal during sinus rhythm. Arrhythmic episodes lasting more than 5 min were aborted using short (1S) pacing bursts of increasing intensity until sinus rhythm was restored. The minimal time between pacing bursts was 1 min. AF inducibility was calculated as the percentage of positive episodes (defined as episodes lasing more than 1S). The total AF duration score evaluated the duration of the AF episodes using a standard scoring scale^39^.

### Echocardiography

Echocardiography measurements were performed with a dedicated system for rodents (Vevo 3100, FUJIFILM VisualSonics, Canada) under lightly anesthesia with 1.5% isoflurane/O2 mixture. Rat were placed in a left decubitus position on a heating pad to maintain a rectal temperature of ~ 37 °C. The duration of the whole echocardiography procedure was restricted to 15 min. 2D images of the left ventricle were taken in parasternal long and short axis views. Long and short axis M-mode images were obtained at the mid papillary muscle area with cursor penetration at the papillary muscle tip^39^. All measurements were averaged for three consecutive cardiac cycles and performed by an experienced technician who was blinded to the treatment groups.

### Histology immunohistochemistry and immunofluorescence analyses

At the terminal procedure, hearts were excised into cold PBS solution. Left atrial (LA) tissue and ventricular short-axis slices were fixed in 4% paraformaldehyde, embedded in paraffin, and sectioned (5 µm thickness), as previously described^39^. Sections were deparaffinized in xylene, rehydrated in a descending alcohol sequence, and brought into distilled water. Masson trichrome (MT) stain was performed according to the manufacturer's protocol (04-010802, Bio-Optica, Milano, Italy). The stained sections were scanned with a panoramic scanner (panoramic MIDI II, 3DHISTH, Budapest, Hungary) and analyzed automatically with customized software (Quant center 2.0 software, 3DHISTH). Five randomly selected images were examined in each section. Myocardial fibrosis was reported as the collagen volume fraction (CVF) and calculated with the equation of total collagen area/total field area. The reader was blinded to group assignment.

TUNEL staining was performed according the manufacturer's protocol (DeadEnd Fluorometric TUNEL System G3250, Promega). Negative controls were done by incubating with the staining solution alone. Positive controls included sections incubated with DNAse I. Following the staining procedure, samples were analyzed for TUNEL-positive cells using a fluorescent microscope (Leica DM2500, Wetzlar Germany). The total number of TUNEL-positive cells in each section was counted and normalized to the surface area of the same section. Analysis was blinded to group assignment.

Immunohistochemical and immunofluorescence staining were performed on the 5 μm-thick sections. Antigen retrieval was performed by 30 min incubation in 0.01 M citrate buffer, pH 6.0 Sections were washed with PBS, pH 7.4, containing 0.1% Triton-X100 (PBST), incubated in 10% NGS for 2 h, followed by overnight incubation at 4 °C with primary antibodies. For immunohistochemical staining, endogenous peroxidase activity was blocked by incubating the sections in 3% H_2_O_2_ for 15 min. Following washing with PBST, sections were incubated for 2 h with the appropriate secondary HRP-conjugated antibodies. The sources and dilutions of primary and secondary antibodies are detailed in Supplemental Table S4. Sections were washed in PBST, and peroxidase activity was visualized by incubating with 3,3-diaminobenzidine (DAB) (ImmPact-DAB, Burlingame, CA). After rinsing in water, the sections were counterstained with hematoxylin, and mounted with mounting medium. The stained sections were scanned with a panoramic scanner (panoramic MIDI II, 3DHISTH, Budapest, Hungary) and analyzed using the IHC Profiler plugin within ImageJ software^41^. Five images from each section of randomly selected areas were examined. The software measured the intensity of the brown color—a HRP reaction product. Only the percentage of “high positive” staining intensity was measured, in order to enhance the accuracy of quantification^42–44^. Data are shown as “fold change” relative to the control group. The reader was blinded to group assignment. Control experiments to exclude non-specific staining by the secondary antibody were carried out using the same protocol without incubation with the primary antibodies (Supplemental Figure S1A). In addition, western blot experiments in which cells were treated with siRNA specific for VDAC1 confirmed the reported specificity of the primary anti-VDAC1 antibodies (Supplemental Figure S1B).

For immunofluorescence, sections were sequentially incubated with the relevant primary and secondary antibodies, as detailed in Table 4S. Next, the tissues were stained with DAPI (0.07 μg/ml). The stained sections were viewed with an Olympus IX81 confocal microscope and analyzed within ImageJ software. Fluorescence intensity of three randomly selected fields from each section were measured. Analysis was blinded to group assignment.

### Statistical analysis

Values are expressed as mean ± SE. Comparison of IHC measurements between two groups was performed using a Mann–Whitney test. Comparison of IHC measurements between the three groups in the human MI microarray (Sort-term, Long-term, Control) was performed by a Kruskal Wallis test with a Dunnett multiple comparison post-test. AF substrate parameters did not have a normal distribution and were, therefore, analyzed using nonparametric testing: Comparisons between 2 and 4 W within each group was done using a Wilcoxon matched-pairs signed rank test. Comparisons between similar weeks in two treatment groups was done using a Mann–Whitney test. The criterion for significance was set at *p* < 0.05. Unless otherwise stated, p-values are displayed graphically as follows: *p < 0.05, **p < 0.01, ***p < 0.001, ns = not significant. For 0.06 ≥ *p* > 0.05, the p-values are indicated in the figures, but results are regarded as non-significant. For the IHC, MT, TUNEL, AF induction, and duration figures, scatter plot graphs show the number of human subjects or rats that were analyzed. For the IF figures, bar graphs represent the number of analyzed fields, and the numbers of animals from which these fields were collected are indicated in the figure legend.

## Results

### VDAC1 overexpression in diseased human cardiac tissues

**Figure 1:**
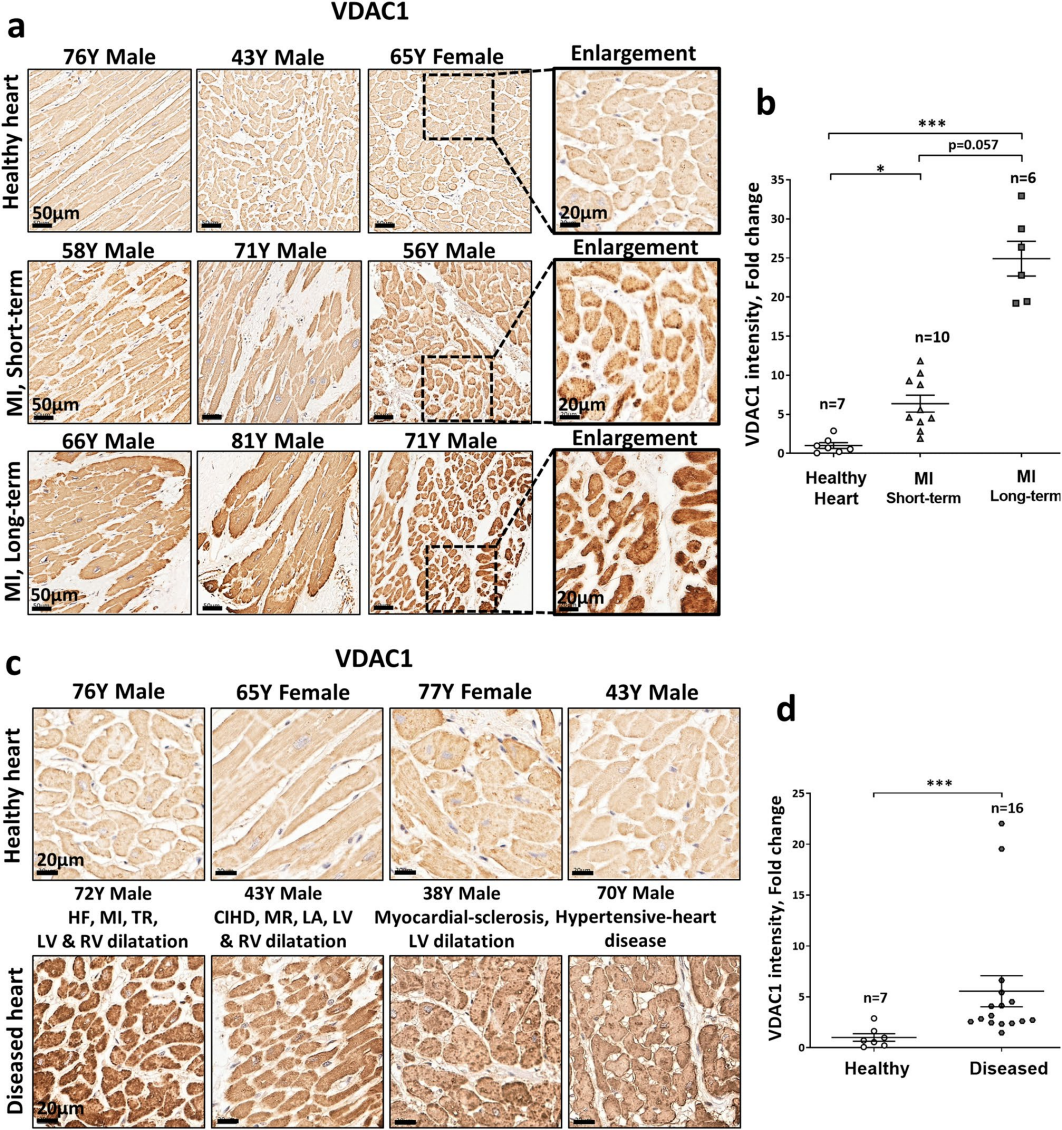
Overexpression of VDAC1 in human cardiac pathologies. (**a**,**b**) VDAC1 IHC staining in the left ventricle (LV) of patients post myocardial infraction (MI) relative to hearts without known cardiac disease. IHC staining presented as fold change relative to the mean intensity of the control samples. “MI short-term” represents samples defined as acute MI or granulation tissue. “MI long-term” represents the samples in which clinical data indicated the presence of scar tissue. Only non-infarcted zones with viable myocardium were analyzed. (**b**) Representative photomicrographs for each condition. Scale bars, 50 µm and enlargement of 20 µm. (**b**) Summarizing scatter plot of quantitative image processing analysis. Note gradual increase in VDAC1 expression levels between MI long-term and MI short-term. Also note, punctuated staining suggesting mitochondrial localization of VDAC1. (**c**,**d**) VDAC1 IHC staining in the LV of patients with cardiac diseases involving dilatation\hypertrophy. HF—Heart failure, TR—tricuspid regurgitation, RV—Right ventricle, CIHD—Chronic ischemic heart disease, MR—Mitral regurgitation. (**c**) Representative photomicrographs for each condition. Scale bars, 20 µm. (**d**) Summarizing scatter plot of quantitative image processing analysis. Note, overall increased expression of VDAC1 in the various pathologies. The number of samples in each group is indicated (n = 6–16).

Analysis of a human cardiac tissue array derived from MI and chronic heart disease patients opened up an opportunity for us to analyze VDAC1 expression levels in the context of one of the most prevalent cardiac pathologies and a leading cause of heart failure and death^45^. Our findings clearly demonstrate increased expression of VDAC1 in the LV following MI as compared to its expression in the LV of non-infarcted individuals. Furthermore, the separation of post-MI samples to MI short-term and MI long-term based on the available clinical data (see “Methods” section for details) indicated increased expression of about sevenfold and 25-fold, respectively, suggesting that VDAC1 overexpression is a gradual process in this clinical context (Fig. 1a,b). It should be mentioned that areas of scar tissue in the MI, long-term samples had negligible staining for VDAC1 and were excluded from our analysis.

**Figure 2:**
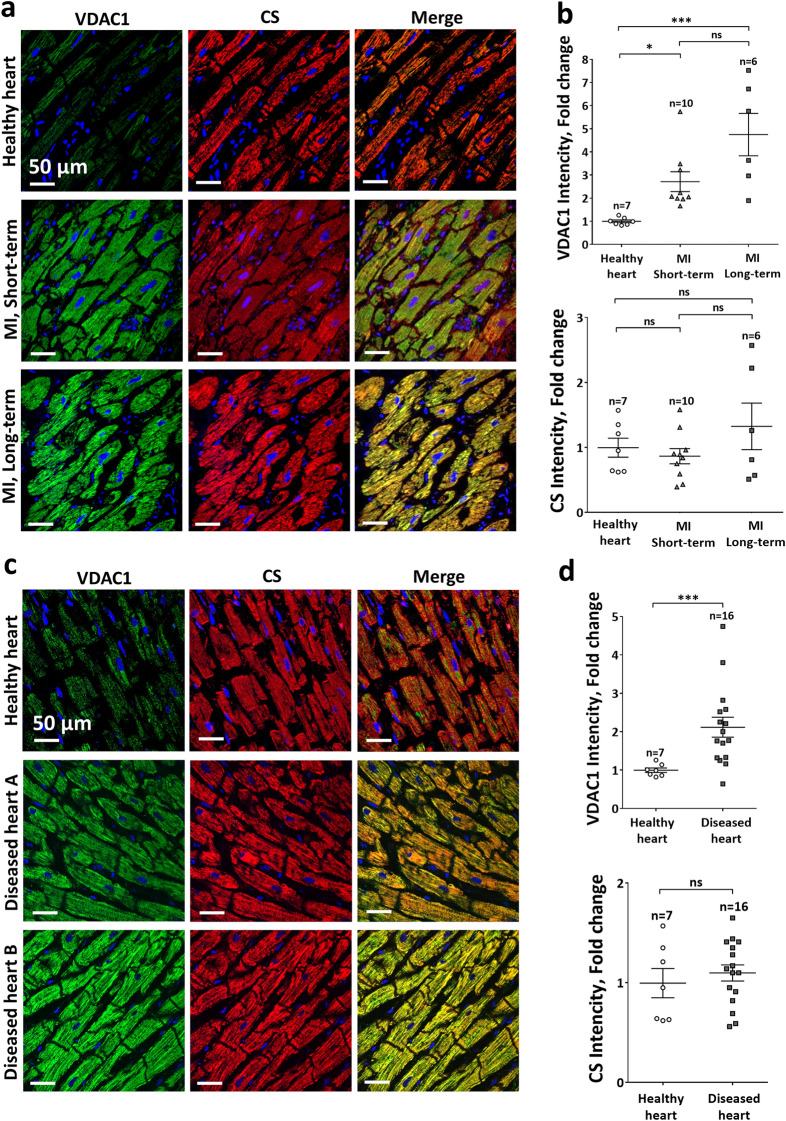
The overexpression of VDAC1 in human cardiac pathologies does not signify changes in mitochondrial mass. Co-immunofluorescent staining of the human cardiac tissue arrays with a VDAC1 specific antibody (ab186321, Table S4) as well as an antibody for citrate synthase (CS) to quantify mitochondrial mass/amount. (**a**,**b**) analysis of the same MI samples as in Fig. 1a,b. (**a**) Representative photomicrographs for each condition demonstrating staining for VDAC1 (green), CS (red) and both (Merged) . Scale bars, 50 µm. (**b**) Summarizing scatter plot of quantitative image processing analysis. (**c**,**d**) analysis of the diseased LV samples as in Fig. 1c,d. (**c**) Representative photomicrographs for each condition demonstrating staining for VDAC1 (green), CS (red) and both (Merged) . Scale bars, 50 µm. (**d**) Summarizing scatter plot of quantitative image processing analysis.

An additional analysis of a tissue array focused on patients reported to have various cardiac abnormalities leading to LV dilatation\dysfunction\hypertrophy (chronic myocardial disease group) also revealed a statistically significant increase in VDAC1 expression levels (Fig. 1c,d). These findings support the notion that cardiac dysfunction is associated with VDAC1 overexpression, although the variability between cases and absence of precise clinical data restricted further analysis of this (see “Discussion” section). Of note, the cases that were present in the tissue array slides were predominantly of male patients. In the MI arrays there were only two females out of sixteen samples. In the diseased heart three out of sixteen were females. Sub-group analysis of the latter group confirmed increased VDAC1 expression levels in the males (Supplemental Figure S2). However, tor the females the number of samples was too small and results were too variable to get to conclusive results. To further confirm the above findings and to additionally exclude the possibility that the increased IHC staining results from increases in mitochondrial density, we independently performed co-immunofluorescent staining of the tissue arrays with another VDAC1 antibody (ab186321, Table 4S) as well as with an antibody against citrate synthase (CS) that is considered as a marker for mitochondrial mass/amount^46^. The results of this analysis nicely replicated the IHC findings of VDAC1 overexpression (Fig. 1) and suggest that VDAC1 was increased per mitochondria and not due to increase in the number of mitochondria (Fig. 2). In addition, since the ab186321 antibody is reported to identify VDAC1 only, these findings suggest that VDAC1 and not another isoform is responsible for the expressional changes.

### VDAC1 overexpression in diseased rat cardiac tissues

**Figure 3:**
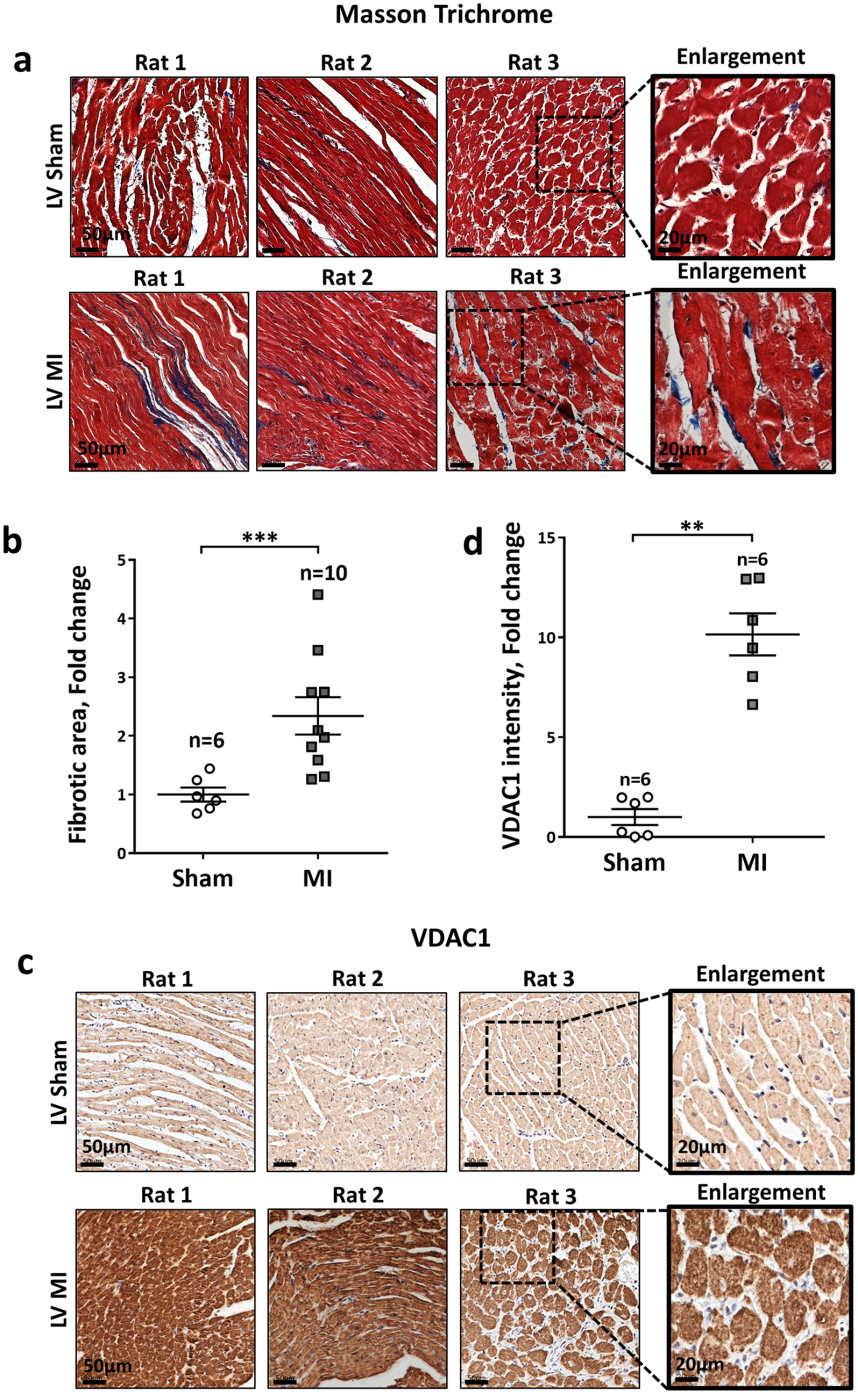
Overexpression of VDAC1 in the LV of rats post MI. (**a**,**b**) Quantitative analysis of myocardial fibrosis in the LV of sham and MI rats using Masson’s trichrome (MT) staining. (**a**) Representative photomicrographs for each condition. Scale bars, 50 µm and enlargement of 20 µm. (**b**) Summarizing scatter plot of quantitative image processing analysis. Note, increased LV fibrosis in MI rats relative to sham, as expected. (**c**,**d**) VDAC1 IHC staining in the LV of MI and sham rats. IHC staining presented as fold change (%) relative to the mean intensity of the sham preparations. In the MI rats, only non-infarcted zones of the LV were analyzed*.* (**c**) Representative photomicrographs for each condition. Scale bars, 50 µm and enlargement of 20 µm. (**d**) Summarizing scatter plot of quantitative image processing analysis. Note increased expression of VDAC1 in LV of MI rats. The number of rats in each group is indicated (n = 6–10).

**Figure 4:**
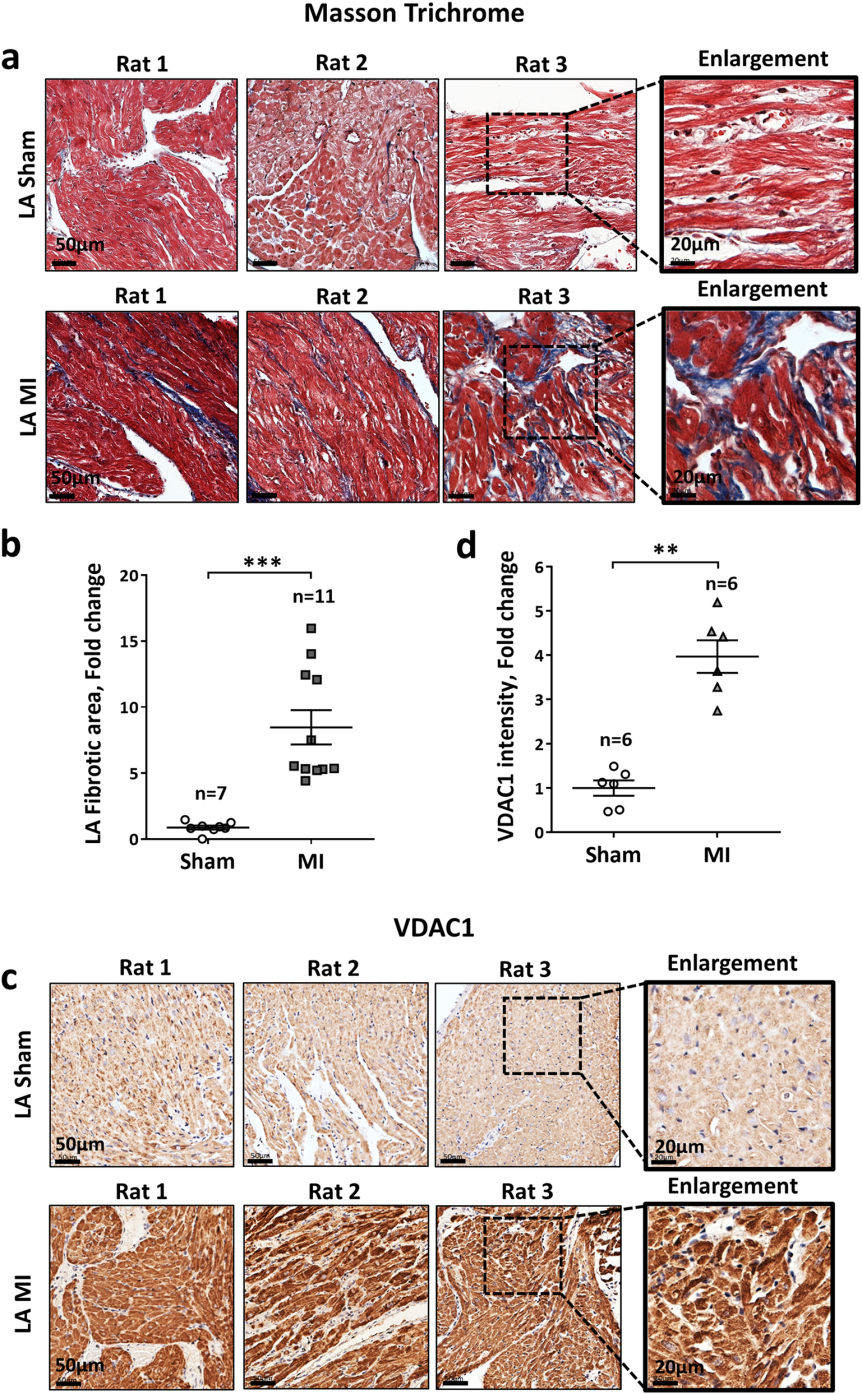
Overexpression of VDAC1 in the LA of rats post MI. (**a**,**b**) Analysis of LA fibrosis (similar representation as in Fig. 2a,b). Note prominent increase of LA fibrosis in MI rats. (**c**,**d**) Analysis of VDAC-1 in the LA (similar representation as in (**c**,**d**). Note increased expression of VDAC1 in LA of MI rats. The number of rats in each group is indicated (n = 6–11).

To substantiate the noted VDAC1 overexpression in human cardiac pathologies, we next evaluated VDAC1 expression levels in rat cardiac tissue samples from our recent study on MI and hyperaldosteronism as triggering factors for AF^39^. As expected, Masson trichrome staining of the LV in rats that were evaluated four weeks post-MI demonstrated prominent fibrosis as a surrogate of structural remodeling (Fig. 3a,b). IHC staining of those samples demonstrated increased expression of VDAC1 with prominent punctuated staining (Fig. 3c,d). Interestingly, rather similar findings including increased atrial fibrosis (Fig. 4a,b), and significant overexpression of VDAC1 (Fig. 4c,d) was observed in the atria of these post–MI rats.

**Figure 5:**
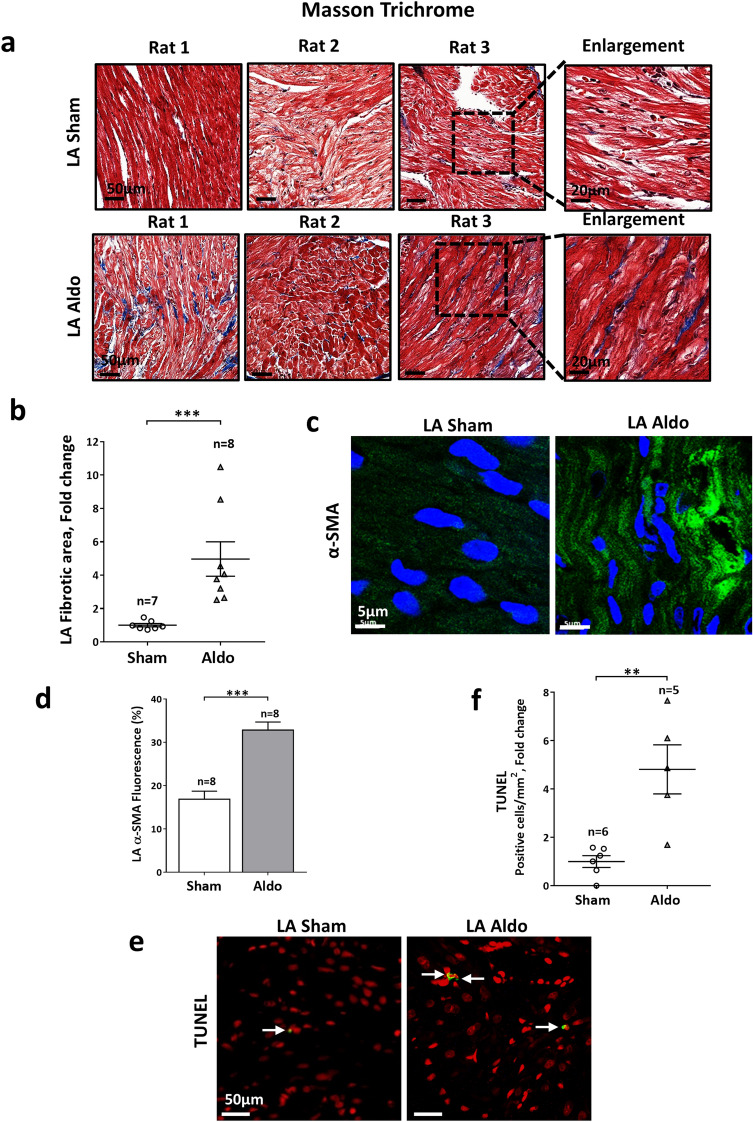
Atrial structural remodeling in rats exposed to excessive Aldo levels. (**a**,**b**) MT staining and its quantitative analysis of myocardial fibrosis in the LA of sham and Aldo rats. (**a**) Representative photomicrographs for each condition. Scale bars, 50 µm and enlargement of 20 µm. (**b**) Summarizing scatter plot of quantitative image processing analysis. Note increased LA fibrosis in Aldo rats. (**c**,**d**) IF staining of the expression of α-SMA in the atrial tissues of the sham and Aldo rats using IF staining. Two fields were analyzed from each rat. n represents the total number fields*.* (**c**)*:* Representative photomicrographs for each condition. Scale bars, 50 µm (**d**) Summarizing scatter plot of quantitative image processing analysis. As expected, increased expression of α-SMA was noted in the Aldo treated atria in accordance with the elevated fibrotic load. (**e**,**f**) TUNEL analysis, performed by counting the total number of TUNEL-positive cells in each LA section normalized to its surface area (**e**) Representative photomicrographs for each condition. Scale bars, 50 µm. (**f**): Summarizing dot plot of quantitative image processing analysis. Note increased apoptosis in the LA of Aldo rats. The number of rats in each group is indicated (n = 5–8).

**Figure 6:**
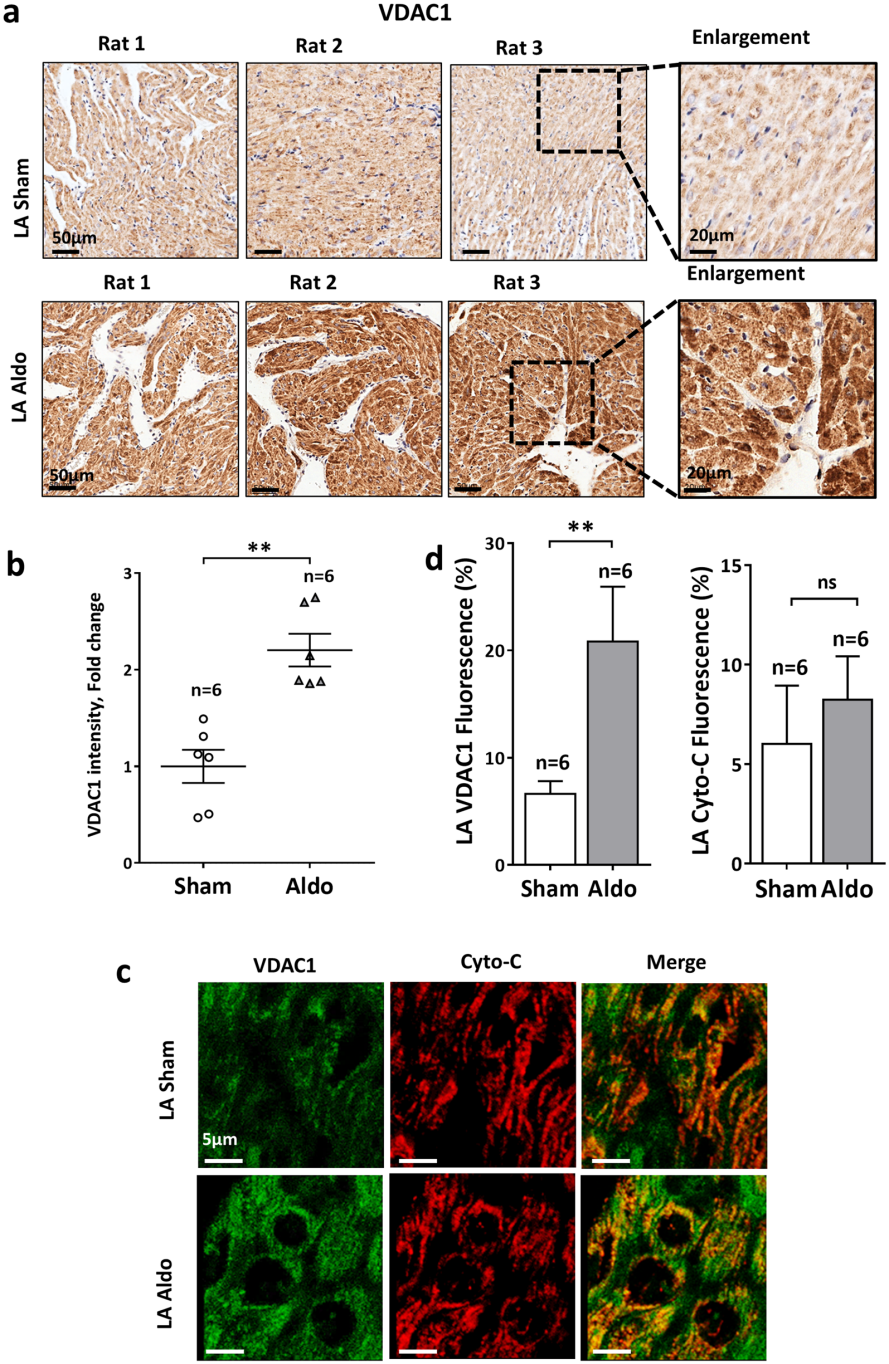
Increased atrial expression of VDAC1 in rats exposed to excessive Aldo levels. (**a**,**b**) VDAC1 expression in the LA evaluated using IHC staining. (**a**) Representative photomicrographs for each condition. (**b**) Summarizing scatter plot of quantitative image processing analysis. Note increased expression of VDAC1 in LA of Aldo rats relative to sham. (**c**,**d**) VDAC1 and Cyto *c* expression in the LA analyzed using IF staining (**c**). (**d**) Quantitative analysis of the IF data. The number of rats in each group is indicated (n = 6).

In contrast to the post-MI rat model, which is characterized by prominent systolic dysfunction and progressive LV remodeling, isolated hyperaldosteronism does not affect the LV systolic function of rats and can mainly lead to atrial structural remodeling in this setting^39,47^. Thus, we decided to test in further detail the possible roles of VDAC1 in the setting of hyperaldosteronism, with a specific focus on the atria. Exposure of rats to Aldo for 4 weeks led to an increase in atrial fibrosis (Fig. 5a,b) with a concomitant increase in α-SMA (Fig. 5c,d) and atrial apoptosis (Fig. 5e,f). Under these conditions, evaluation of VDAC1 expression levels by both IHC and IF staining revealed marked overexpression (Fig. 6). IHC staining of VDAC1 revealed a punctuated appearance of VDAC1, as expected for mitochondrial localization. IF co-staining for VDAC1 and the Cyto *c* demonstrated mitochondria co-localization (Fig. 6c). Interestingly, the overexpressed VDAC1 in the presence of Aldo was not fully localized to the mitochondria. This may result from the localization of the overexpressed VDAC1 in compartments other than the mitochondria, as we have shown in the skeletal muscle sarcoplasmic reticulum, endoplasmic reticulum of cerebellar cells and in the plasma membrane of β-cells in T2D^15,48,49^. The increased VDAC1 expression levels were not due to an increase in mitochondrial number, as indicated by the absence of change in the expression levels of Cyto *c* in the Aldo rats (Fig. 6c,d). Surprisingly, increased expression of VDAC1 was also found in the LV of the Aldo rats, regardless of the fact that these rats had normal LV function^39^ and normal LV fibrotic tissue levels (Supplemental Figure S3).

### Effects of VBIT-4 treatment in rats with hyperaldosteronism

**Figure 7:**
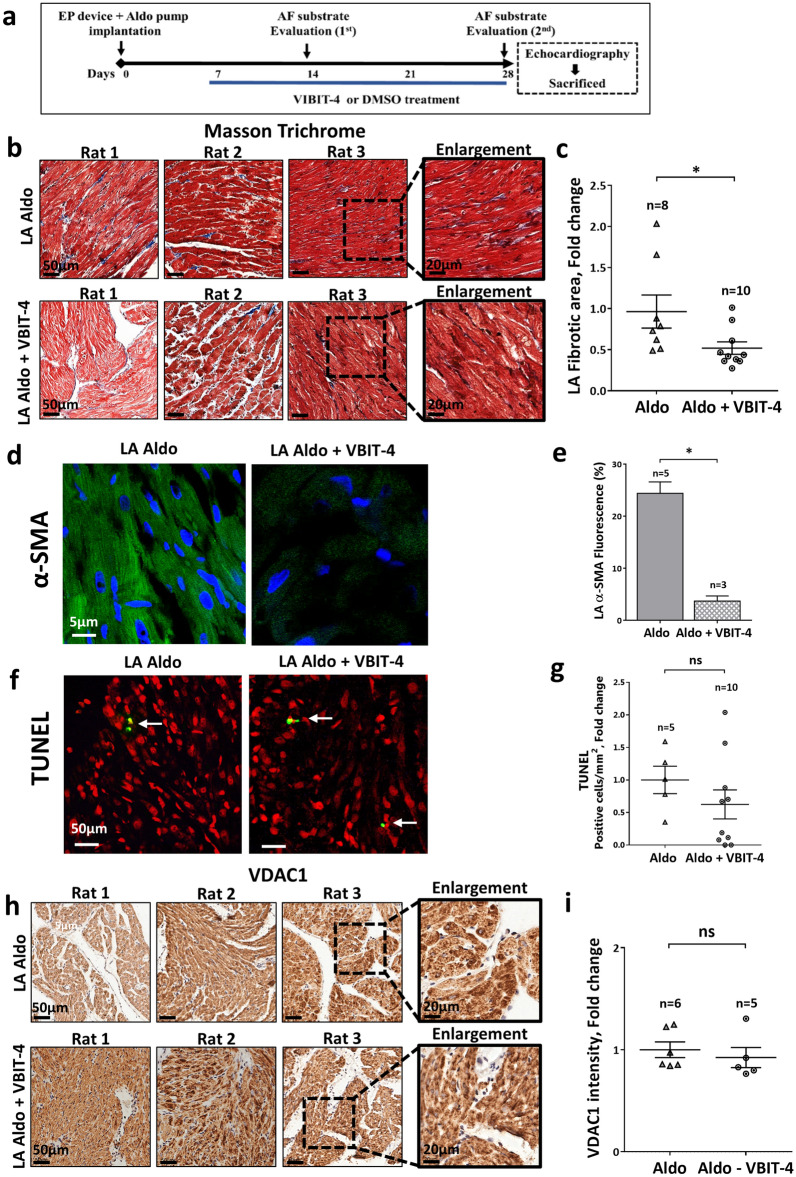
Attenuating effect of VBIT-4 on atrial fibrosis induced by hyperaldosteronism. (**a**) Schematic outline of the interventional study of VBIT-4 (see “Methods” section for technical details of the experimental protocol). (**b**,**c**) MT staining and quantitative analysis of myocardial fibrosis in the LA of Aldo and Aldo + VBIT-4-treated rats. (**b**) Representative photomicrographs for each condition. Scale bars, 50 µm and enlargement 20 µm. (**c**) Summarizing scatter plot of quantitative image processing analysis. Note a decrease in LA fibrotic area following VBIT-4 treatment. (**d**,**e**) IF staining of α-SMA expression in the atrial tissues of the sham and Aldo rats*.* (**d**) Representative photomicrographs for each condition. Scale bars, 5 µm. (**e**): Quantitative analysis of image expressing α-SMA. (**f**,**g**) TUNEL staining analysis (as in Fig. 4) in LA tissues of Aldo and Aldo + VBIT-4 treated rats. (**f**) Representative photomicrographs for each condition. Scale bars, 50 µm. (**g**) Summarizing scatter plot of quantitative image processing analysis. (**h**,**i**) VDAC1 IHC staining intensity in the LA. (**h**) Representative photomicrographs for each condition. Scale bars, 50 µm and enlargement 20 µm. (**i**) Summarizing scatter plot of quantitative image processing analysis. The number of rats in each group is indicated (n = 5–10).

As previously reported for studies involving mice^17,18^, VBIT-4 treatment administered through the drinking water is well tolerated by hyper-aldosteromenic rats. The final body weight of the VBIT-4-treated group was similar to the control group, and there were no differences in cardiac echocardiographic parameters (Supplemental Table S5). In the atria, the VBIT-4 treatment protocol (Fig. 7a) markedly reduced the level of fibrosis (Fig. 7b–e). A mild tendency of reduction in apoptotic staining was also noted, but results were too variable, and this tendency did not reach statistical significance (Fig. 7f,g). VBIT-4 did not affect the expression levels of VDAC1 or Cyto *c* (Fig. 7h,i; Supplemental Figure S4). Surprisingly, VBIT-4 also reduced the presumably normal LV fibrosis levels (Supplemental Figure S5a,b). As in the atria, the VDAC1 expression level in the LV was unaffected by VIBIT-4 treatment (Supplemental Figure S5c,d).

In regard to the AF burden, we recently reported marked variability specifically in the presence of hyperaldosteronism^39^. Thus, although VBIT-4 treatment seemed to somewhat attenuate the AF induction and total AF duration score at 4 weeks compared to 2 weeks, the results overall were too variable to reach a significant level with the number of rats we used in the current pilot study (Supplemental Figure S6).

## Discussion

The present study substantiates previous evidence in the literature regarding the possible involvement of VDAC1 in common cardiac pathologies^24,30–33,37^. Our study demonstrated increased levels of VDAC1 in the post-MI setup, as well as in the LV of patients with chronic cardiac dilatation, hypertrophy, and dysfunction. VDAC1 overexpression was also found in the LA and LV of rats exposed to MI or excessive aldosterone levels. Finally, the study demonstrated that treatment with the VDAC1 oligomerization apoptosis and mitochondria dysfunction inhibitor, VBIT-4, reduces atrial fibrosis in the setting of hyperaldosteronism. These main findings are discussed below.

Our analysis of IHC VDAC1 staining data in human cardiac tissue array samples enabled quantification of VDAC1 expression levels in the LV of human patients with cardiac pathology. Although the clinical data related to these samples were limited, they could still be used to group the samples in a logical way, perform statistical analysis, and reach important conclusions. Our findings support the notion that both MI and chronic heart disease associated with LV dilatation markedly increase VDAC1 expression levels in the LV myocardium. Conformation of these findings using co-immunostaining for VDAC1 and citrate synthase indicated that these results do not express a non-specific change in mitochondrial mass\density, but rather an increase in VDAC expression levels per mitochondrion. The findings in rats further support this idea and indicate that both MI and hyperaldosteronism can increase VDAC1 levels not only in the ventricular tissue, but also in the atria. The comparison between MI short-term and MI long-term in the human data revealed a tendency for time-dependent increase in VDAC1 with higher expression in the chronic phase. This finding was noted independently with both IHC and immunostaining. With both methods post-hoc differences from the controls were of higher significance in the long-term samples. However, pos-hoc differences between short-term and long-term groups did not reach statistical significance and therefore this observation should be interpreted with caution. Interestingly, in a previous study by Mitra et al. (2013)^24^, VDAC1 upregulation was not detected in rats 10 days post-MI. However, that study did not examine later stages of MI as in our current study. Thus, it is possible that overexpression of VDAC1 in the rat model is also time dependent and reaches prominent levels only at later stages when LV remodeling become excessive. Our ventricular findings in patients with chronic heart disease, as well as in rats with hyperaldosteronism, are in line with the documentation of marked VDAC1 overexpression in rats subjected to hypertrophy by renal artery ligation^24^. RAAS activation is a main consequence of renal artery ligation, as well as a well-known trigger of cardiac remodeling in patients with cardiac dysfunction^50^. Thus, it is conceivable that RAAS activation is a key factor in the initiation of VDAC1 overexpression. The fact that hyperaldosteronism per se could trigger a global increase of VDAC1 in the heart focuses specific attention to the possible role of aldosterone as the main trigger for VDAC1 overexpression in heart disease. Interestingly, RAAS activation also has a known role in a rat model of diabetic cardiomyopathy, in which increased VDAC1 levels were also recently documented^51^. In this model, VDAC1 expression was linked to the H19/miR-675 axis. Thus, in continuation with our current findings, it would be interesting to evaluate whether aldosterone might affect this axis leading to VDAC1 overexpression.

Circulating or locally produced aldosterone has been shown to stimulate cardiac collagen synthesis and fibroblast proliferation, as well as acceleration of cardiomyocytes apoptosis^29,52^. Indeed, our current analysis in rats following chronic exposure to excessive aldosterone levels demonstrated an increase of both atrial fibrosis and apoptosis. Increased expression of VDAC1 in this pathological setting might be suggested to occur either as a maladaptive process or as a compensatory mechanism against the detrimental effects of aldosterone. The decreased atrial fibrosis that we documented following VBIT-4 treatment (discussed below) strongly supports the maladaptive option. Further studies are required to elaborate the relation between aldosterone levels and VDAC1 expression and to define how VDAC1 might be mechanistically involved in aldosterone-induced detrimental effects in the heart in general and specifically in the atria.

The interventional part of our study indicates that VBIT-4 can decrease atrial fibrosis in rats exposed to excessive levels of aldosterone. Fibrosis is a hallmark in various myocardial pathologies. Furthermore, novel experimental approaches targeting activated cardiac fibroblasts are considered to be promising potential therapies for cardiac diseases including atrial fibrillation^53,54^.

Apoptosis has been identified as a key player in the initiation and propagation of cardiac fibrosis^29,55,56^, considering the limited regeneration capacity of the cardiac myocyte^57^. By its function as an inhibitor of VDAC1 oligomerization, apoptosis, and preventing mitochondria dysfunction, VBIT-4 can target early stage of apoptotic cell death in a potent and effective manner without an effect of VDAC1 expression, as expected according to its proposed mode of action^17,18^. Thus, preventing aldosterone from promoting atrial fibrosis by VBIT-4 treatment seems logical. However, we could not directly demonstrate a reduction in atrial apoptosis. One option to explain this apparent discrepancy is the rather low and variable degree or extent of apoptosis that we detected in our atrial preparations, which could have limited the ability to reach significance in the current experimental settings. Alternatively, considering VBIT-4 effects in preventing mitochondria dysfunction, ROS production, and restoring cellular Ca^2+^ levels, it is possible that VBIT-4 can directly inhibit cardiac fibrosis in a manner that is independent of its anti-apoptotic effect, but protective against mitochondria dysfunction. The latter option may be supported by the ability of VBIT-4 to inhibit the apparently normal fibrotic load of the ventricular myocardium. Of note, owing to the relative abundance of Cyto c within cells the use of confocal microscopy to distinguish between mitochondrial and cytosolic Cyto c is not easy, particularly if only small fraction of the Cyto c is released. Considering this and the relatively low level of apoptosis induced by Aldo (Fig. 5), it is not surprising that we did not identify VBIT-4 inhibition of Cyto c release. Interestingly, VIBIT-4 was recently found to prevent palmitate-induced cardiomyocyte dysfunction as well as mPTP opening and death^58^, further supporting the possible therapeutic role of this compound in stress conditions leading to myocardial dysfunction and apoptotic cell death. Our current findings should pave the way to further studies with pathological triggers leading to more prominent cardiac apoptosis and fibrosis such as trans-aortic constriction or MI, which may clearly dissect between the two noted above options.

The electrophysiological part of our study indicated that although VBIT-4 reduced atrial fibrosis, it did not decrease AF induction and duration in the current setting. Interestingly, although fibrotic load was suggested to have a critical role in the pathogenesis of AF in rats exposed to excessive aldosterone levels^47^, our recent study demonstrated no correlation between the fibrotic load and the AF load in the presence of hyperaldosteronism while such correlation was clearly noted in rats exposed to MI^39^. Thus, it is possible that the electrophysiological effects of aldosterone are more important in triggering AF substrate in this model, as suggested by Lammers et al. (2012)^59^.

Overall, our human and rat findings support the notion that VDAC1 overexpression can have major detrimental roles in the myocardium under common pathological settings, such as post-MI, and LV dilatation\dysfunction, possibly through RAAS activation and increased aldosterone levels. Our findings also indicate that pharmacological inhibition of VDAC1 activity can have beneficial effects in preventing the formation of fibrosis in the diseased myocardium and, thus, may serve as a new therapeutic option in various cardiac pathologies that are characterized by excessive myocardial fibrosis.

## Supplementary Information

Supplementary Information.
