# Supplementary material for: VDAC1 in the diseased myocardium and the effect of VDAC1-interacting compound on atrial fibrosis induced by hyperaldosteronism
Source: Sci Rep. 2020 Dec 16;10:22101. doi: 10.1038/s41598-020-79056-w (PMC7744539; doi:10.1038/s41598-020-79056-w)
Supplement: Supplementary file 1 — Supplementary Information. [file 41598_2020_79056_MOESM1_ESM.docx]

**SUPPLEMENTAL MATERIAL**

**VDAC1 in the diseased myocardium and the effect of VDAC1-interacting compound on atrial fibrosis induced by hyperaldosteronism**

Hadar Klapper-Goldstein^1,2^, Ankit Verma^3,4^, Sigal Elyagon^1,2^, Roni Gillis^1,2^, Michael Murninkas^1,2^, Srinivas Pittala^3,4^, Avijit Paul^3,4^, Varda Shoshan-Barmatz,^3,4#^ and Yoram Etzion^1,2#^

**Affiliations:**

^1^Cardiac Arrhythmia Research Laboratory, Department of Physiology and Cell Biology, Faculty of Health Sciences, Ben-Gurion University of the Negev, Beer Sheva, Israel

^2^Regenerative Medicine & Stem Cell Research Center, Ben-Gurion University of the Negev, Beer Sheva, Israel

^3^Department of Life Sciences, Ben-Gurion University of the Negev, Beer Sheva, Israel

^4^National Institute for Biotechnology in the Negev, Ben-Gurion University of the Negev, Beer Sheva, Israel

**Address for Correspondence:** Yoram Etzion. Cardiac Arrhythmia Research Laboratory, Department of Physiology and Cell Biology, Faculty of Health Sciences, Ben-Gurion University of the Negev. P.O. Box 653, Beer Sheva 84105, Israel. Tel: +972-8-647-9901, Fax: +972-8-647-9875; Email: tzion@bgu.ac.il

**Supplemental methods**

**VDAC1 siRNA in A549 cells**: The cell culture was maintained in DMEM culture medium supplemented with 10% FBS 1 mM L-glutamine, 100 U/ml penicillin, and 100 μg/ml streptomycin. at 37°C and 5% CO2. Cells were seeded (100,000 cells/well) in 6-well culture dishes to 40-60% confluence and transfected with the indicated si-hVDAC1 concentration using the JetPRIME transfection reagent (Illkirch, France), according to the manufacturer’s instructions. Si-RNA scrambled served as control. 24 h post-transfection, cells were harvested and western blot analysis was performed using anti-VDAC1 antibody (Abcam, ab15895, 1:5000).

**Supplementary Figures**

**
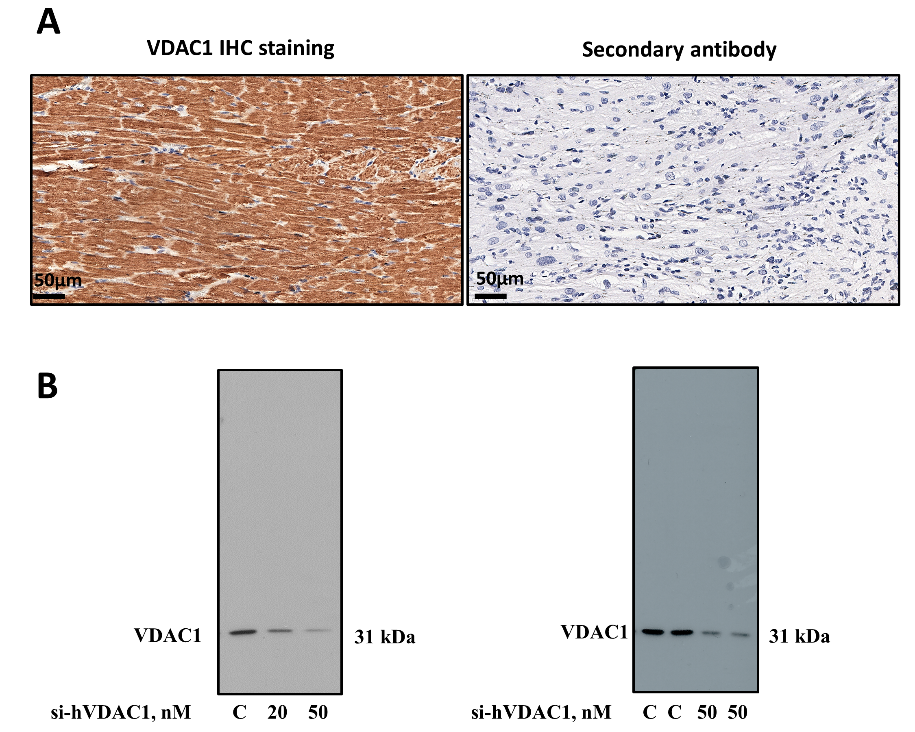
**

**Figure S1: Specificity of anti-VDAC1 antibody (Abcam, ab15895). *A.*** *Left*: Representative VDAC1 IHC staining of human LV myocardium and *Right:* negative control, staining of the same tissue carried out without incubation with primary anti-VDAC1 antibodies. Note the dependence of the IHC staining on incubation with the anti-VDAC1 antibody). ***B.*** Western blot analysis of A549 cell lysates treated with siRNA against VDAC1 (si-hVDAC1). Si-RNA scrambled served as control (C). Right and Left, two independent experiments. Note the appearance of a single band at 31 kDa, which is markedly receded by the si-hVDAC1. Both Coomassie blue and Ponceau staining confirmed similar loading in all lanes (not shown).


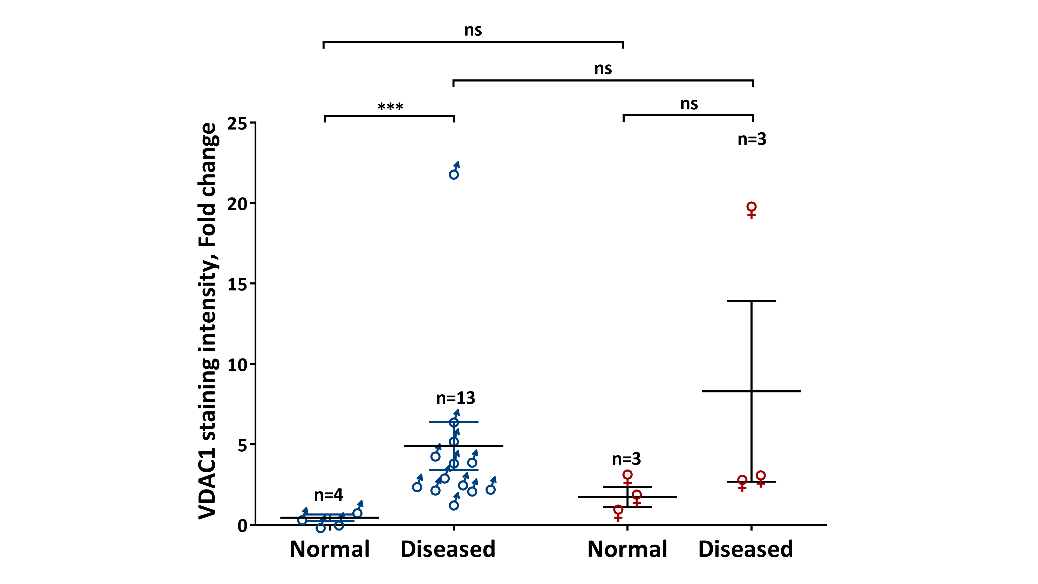


**Figure S2:** **Gender sub analysis of the VDAC1 IHC data**: Analysis of the data in Figure 1D based on gender. Note that the majority of patients were males and the data for males was conclusive in this sub analysis. For females, cases were too few and results were too variable to get conclusive results.

**
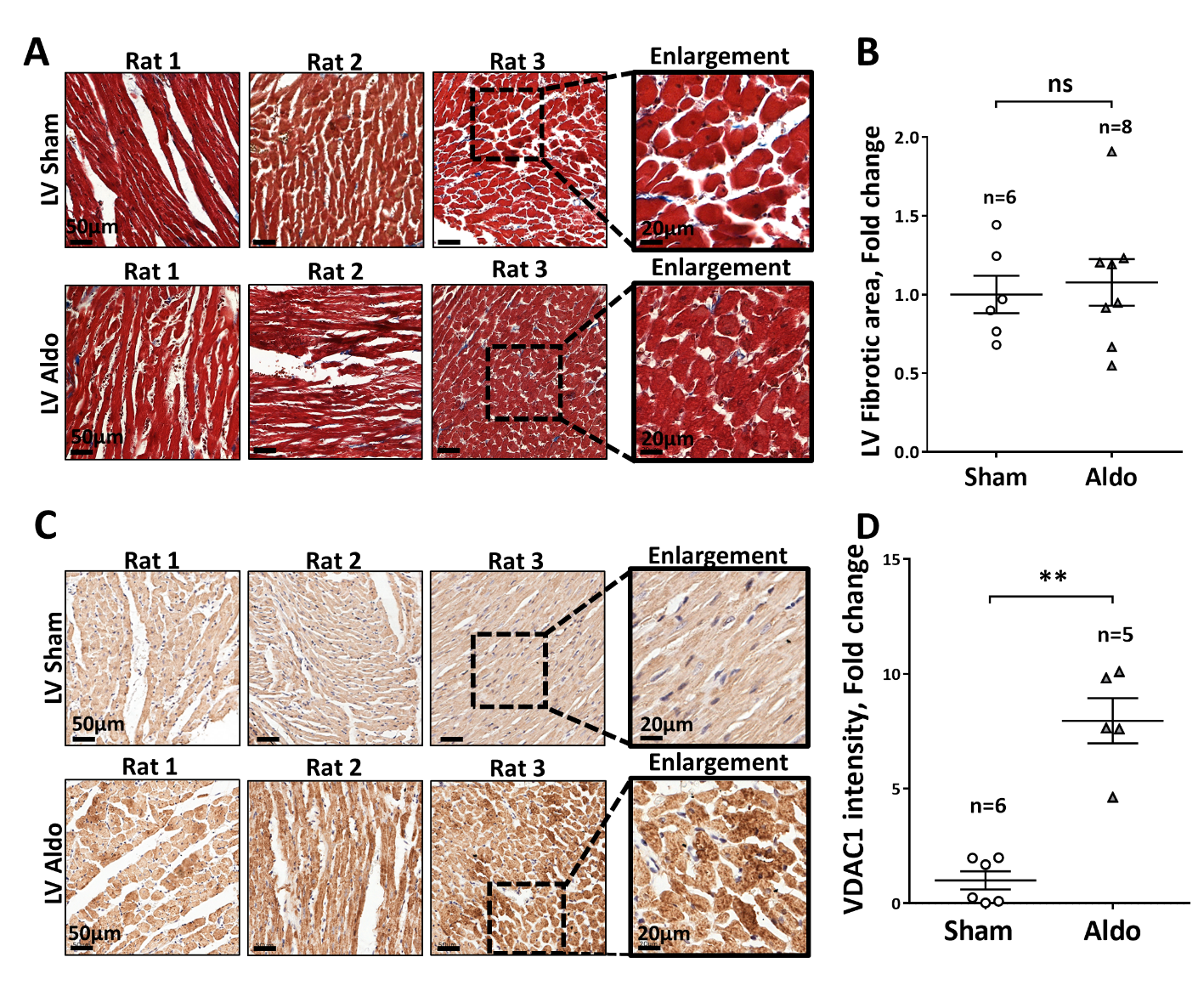
**

**Figure S3: Increased expression of VDAC1 with no increase in fibrosis in the LV of Aldo rats.**

**(A,B)** Quantitative analysis of myocardial fibrosis in the LV of sham and Aldo rats (Masson Trichrome staining). ***A***: Representative photomicrographs for each condition. Scale bars, 50µm and enlargement 20µm ***B***: Summarizing scatter plot of quantitative image processing analysis. Note no differences in LV fibrosis in Aldo rats relative to sham. **(C,D)** VDAC1 IHC staining intensity in the LV. ***C****:* Representative photomicrographs for each condition. ***D***: Summarizing scatter plot of quantitative image processing analysis. Note increased expression of VDAC1 in LV of Aldo treated rats relative to sham. The number of tissue samples in each group is indicated (n= 5-8).

**
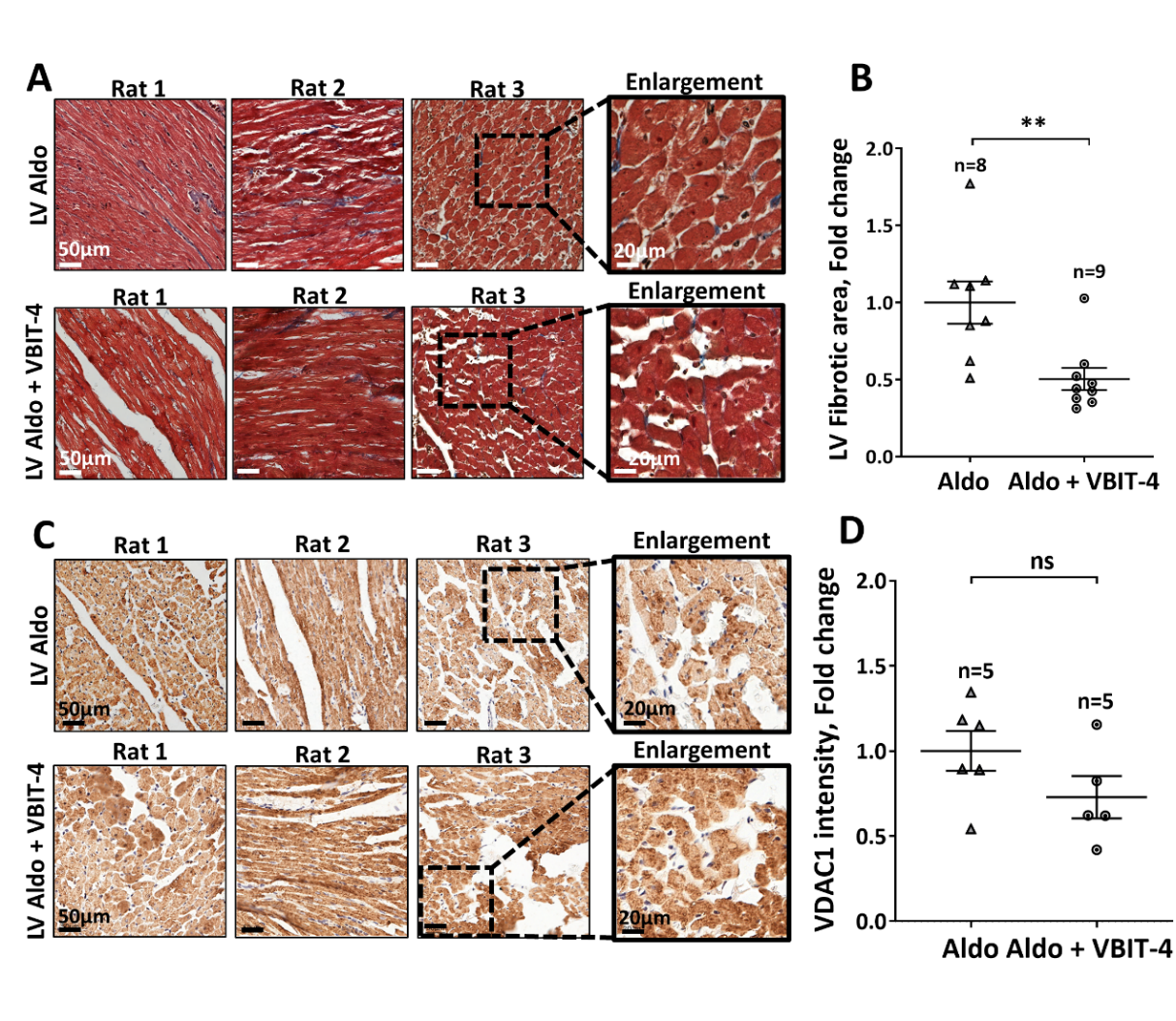

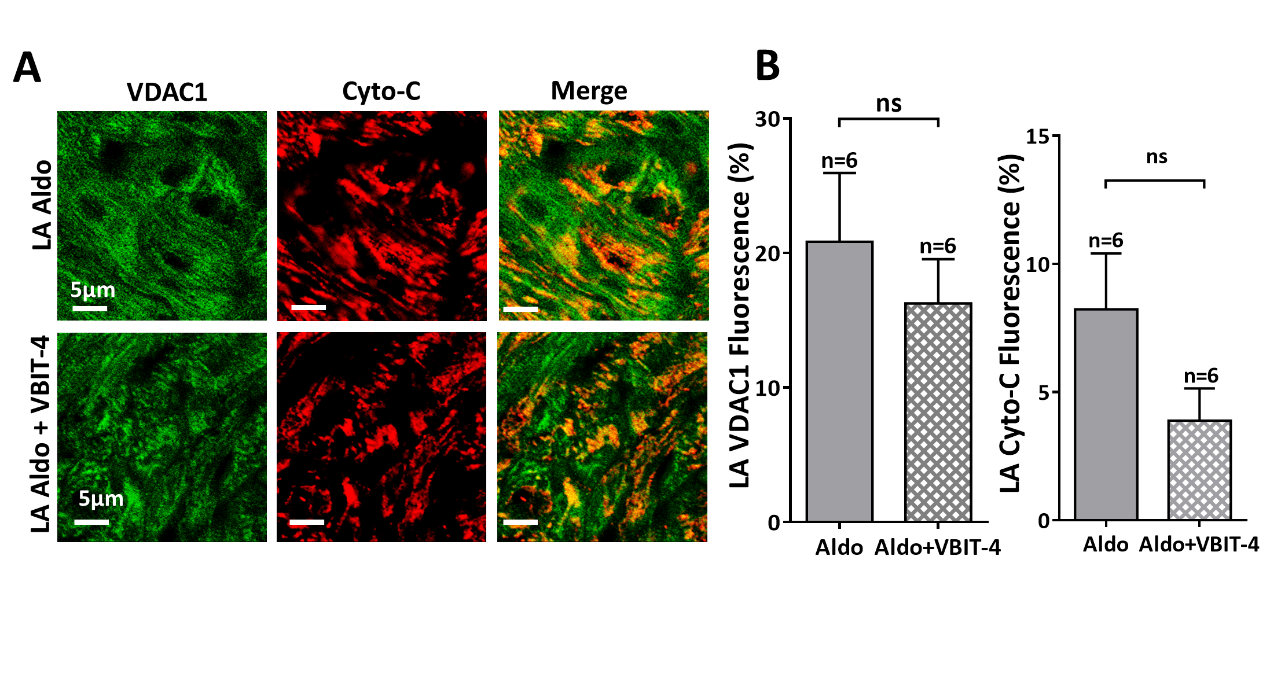
Figure S4: VDAC1 and Cytochrome *c* levels were not affected by VBIT-4 treatment.** Immunofluorescence staining for VDAC1 and Cyto *c* in the LA of Aldo and Aldo+VBIT-4 treated rats. Two fields were analyzed from each rat tissue. n represents the total number of fields*.* ***A****:* Representative photomicrographs. ***B****:* Summarizing scatter plots of quantitative image processing analysis.

**Figure S5: VBIT-4 decreased the LV fibrotic load of Aldo rats. (A,B)** Quantitative analysis of myocardial fibrosis in the LV tissue sections of Aldo and Aldo+VBIT-4 treated rats (MT staining). ***A***: Representative photomicrographs for each condition. Scale bars, 50µm and enlargement 20µm. ***B***: Summarizing scatter plot of quantitative image processing analysis. Note a decrease in LV fibrotic load following VBIT-4 treatment. **(C,D)** IHC staining of VDAC1 in the LV of the Aldo and Aldo+VBIT-4 treated rats*.* ***C****:* Representative photomicrographs for each condition. ***D***: Summarizing scatter plot of quantitative image processing analysis. Scale bars, 50µm and enlargement of 20µm. Note no differences in VDAC1 expression in LV following VBIT-4 treatment. The number of rats in each group is indicated (n= 5–9).

**
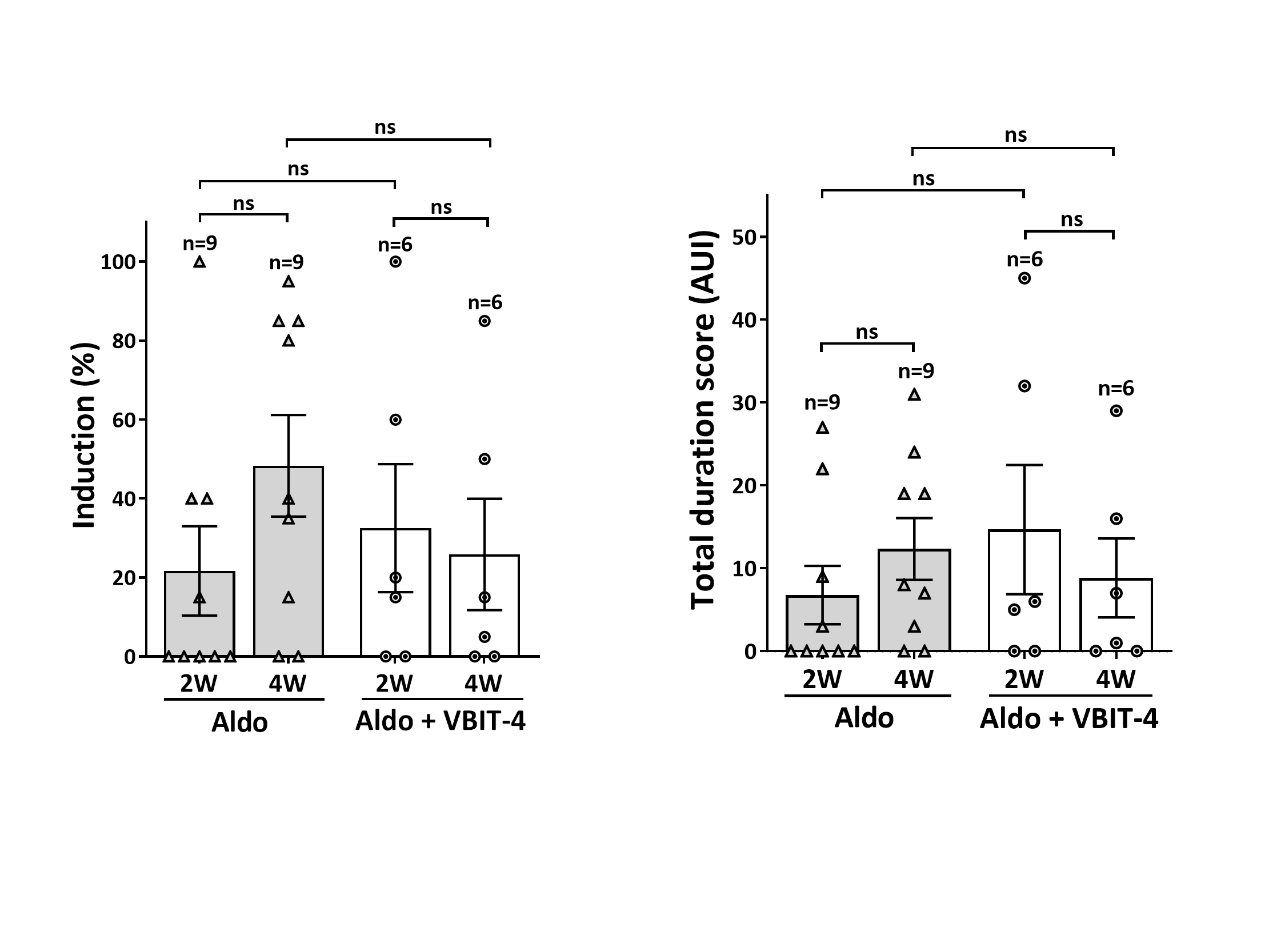
**

**Figure S6:** **VBIT-4 treatment had no effect on atrial fibrillation substrate. *A:*** Atrial fibrillation (AF) inducibility and ***B:*** Total AF duration score (see Methods section for details) of Aldo and Aldo+VBIT-4 treated rats using our standard testing protocol [1]. Comparison of AF inducibility and AF duration score between Aldo and Aldo+VBIT-4 rats at same time frame (2W or 4W) was performed using a Mann-Whitney test. Comparison of AF inducibility and AF duration score between 2W and 4W within each group was performed with a Wilcoxon test. ns = not significant. Note no differences in AF substrate following VBIT-4 treatment in both 2W and 4W. The number of rats in each group is indicated (n= 6–9). Aldo treatment by itself was previously shown to increase both AF induction and AF duration score compared to control animals tested 1W post implantation [1].

**Supplementary Tables:**

**Table S1**: Details of MI-patients included in the analysis of Figure 1A,B

|  | Patient | | Tissue | | MI timing |
| --- | --- | --- | --- | --- | --- |
|  | Gender | Age | Diagnosis | Location | Short\Long term |
| 1 | F | 49 | Acute infraction | LV Post | short-term |
| 2 | M | 65 | Acute infraction | LV Sept | short-term |
| 3 | M | 56 | Acute infraction | LV Sept | short-term |
| 4 | M | 81 | Acute infraction | LV Sept | short-term |
| 5 | M | 58 | Acute infraction | LV Post | short-term |
| 6 | M | 57 | Acute infraction | LV Post | short-term |
| 7 | F | 71 | Granulation tissue | LV Ant | short-term |
| 8 | M | 89 | Granulation tissue | LV Ant | short-term |
| 9 | M | 60 | Granulation tissue | LV Post | short-term |
| 10 | M | 71 | Granulation tissue | LV Post | short-term |
| 11 | M | 67 | Myocardial scar | LV Sept | Long-term |
| 12 | M | 71 | Myocardial scar | LV Sept | Long-term |
| 13 | M | 59 | Myocardial scar | LV Sept | Long-term |
| 14 | M | 81 | Myocardial scar | LV Post | Long-term |
| 15 | M | 60 | Myocardial scar | LV Ant | Long-term |
| 16 | M | 66 | Myocardial scar | LV Post | Long-term |

Cardiovascular Tissue Microarray Human Myocardial Infarction (Cat.-No.: 401 4101) was obtained from Provitro AG (Charitéplatz 1, 10117 Berlin, Germany).

Abbreviations:

LV–Left ventricle

Ant–Anterior

Post–Posterior

Sept–Septal

**Table S2**: Details of cardiac disease patients included in the analysis of Figure 1C, D

Cardiovascular Tissue Microarray Human Heart Tissue, Myocardial Hypertrophy I (Cat.-No.: 401 4102) was obtained from Provitro AG (Charitéplatz 1, 10117 Berlin, Germany).

|  | Patient | | Tissue | |
| --- | --- | --- | --- | --- |
|  | Gender | Age | Diagnosis | Location |
| 1 | M | 80 | Fibrosis and lipomatosis of the left ventricle | LV Sept |
| 2 | M | 62 | Myocardial infarctions, dilatation of both ventricles, coronary heart disease, arrhythmia, 4-fold bypass | LV Sept |
| 3 | M | 54 | Dilatation of left ventricle, calcification of the base of mitral valve | LV Sept |
| 4 | M | 76 | Dilatation of left ventricle with rounded apex cordis | LV Sept |
| 5 | M | 62 | Cardiogenic shock, aortal valve replacement, 3-fold coronary bypass, myocardial infarction (ventral left ventricle) | LV NSL |
| 6 | M | 70 | Hypertensive heart disease | LV NSL |
| 7 | M | 43 | Decompensated chronic ischemic heart disease, dilatation of left atrium, left and right ventricles, Mitral valve insufficiency | LV NSL |
| 8 | M | 63 | Dilatation and lipomatous transformation of left ventricle, calcification of mitral and aortic valves | LV NSL |
| 9 | M | 68 | Dilatation of left and right ventricles | LV NSL |
| 10 | M | 66 | Chronic ischemic heart disease, infarction of ventral left ventricle, dilatation of both ventricles | LV NSL |
| 11 | M | 72 | Cardiac failure, Ischemia, myocardial infarction of posterior left ventricle, dilatation of both ventricles, tricuspid valve insufficiency | LV NSL |
| 12 | M | 38 | Myocardial sclerosis and dilatation of left ventricle | LV NSL |
| 13 | F | 52 | Chronic Cor pulmonale, myocardial sclerosis of left ventricle, dilatation of right ventricle | LV Sept |
| 14 | F | 65 | Dilatation of both ventricles | LV Sept |
| 15 | F | 76 | Hypertensive heart disease, myocardial infarction of left posterior ventricle, lipomatosis of left ventricle, dilatation of right ventricle | LV NSL |
| 16 | F | 53 | Myocardial infarction with acute reinfarction of left ventricle (anterior,  posterior, and septum), dilatation of both ventricles | LV NSL |

Abbreviations:

LV – Left ventricle

Sept – Septal

NSL– Non-specific localization

**Table S3**: Details of healthy patients included in the analysis of Figure 1A–D

|  | Patient | | Tissue | |
| --- | --- | --- | --- | --- |
|  | Gender | Age | Diagnosis | Location |
| 1 | M | 69 | Normal tissue | LV NSL |
| 2 | F | 77 | Normal tissue | LV NSL |
| 3 | F | 70 | Normal tissue | LV NSL |
| 4 | F | 57 | Normal tissue | LV NSL |
| 5 | M | 62 | Normal | LV NSL |
| 6 | M | 64 | Normal | LV NSL |
| 7 | M | 84 | Normal | LV NSL |

Cardiovascular Tissue Microarray Human Myocardial Infarction (Cat.-No.: 401 4101) and Human Left Heart Tissue, Myocardial Hypertrophy I (Cat.-No.: 401 4102) were obtained from Provitro AG (Charitéplatz 1, 10117 Berlin, Germany).

Abbreviations:

LV– Left ventricle

NSL– Non-specific localization

**Table S4**: Details of antibodies, source, and dilution used in the study

| **Antibody** | **Source and Catalog No.** | **Dilution IHC, IF** |
| --- | --- | --- |
| Donkey anti-Mouse HRP conjugated | Abcam, Cambridge, UK, ab98799 | 1:500 |
| Goat anti-Rabbit HRP conjugated | Promega, Madison, WI, W401B | 1:500 |
| Rabbit polyclonal anti-VDAC1 | Abcam, Cambridge, UK, ab15895 | 1:500 |
| Mouse monoclonal anti-VDAC1 | Abcam, Cambridge, UK, ab186321 | 1:400 |
| Mouse monoclonal anti-Cytochrome C | Biosciences, CA, USA, bd556432 | 1:300 |
| Rabbit polyclonal anti-α-SMA | Abcam, Cambridge, UK, ab5694 | 1:300 |
| Rabbit polyclonal anti-Citrate synthetase | Abcam, Cambridge, UK, ab96600 | 1:300 |
| Goat anti-Rabbit Alexa flour 488 | Abcam, Cambridge, UK, a1108 | 1:1000 |
| Goat anti-Mouse Alexa flour 568 | Abcam, Cambridge, UK, a11004 | 1:750 |

**Table S5**: Echocardiography measurements of Aldo and Aldo+VBIT-4 treated groups

|  | **Sham *** | **Aldo** | **Aldo+VBIT-4** | **p-value** |
| --- | --- | --- | --- | --- |
| Body Weight (g) | 416.3±3.75 | 426.3±10.73 | 422.3±13.27 | ns |
| Heart rate (b.p.m) | 344.5±3.3 | 310.3±9.05 | 314.0±16.62 | ns |
| LVIDd (mm) | 8.41±0.18 | 8.49±0.28 | 8.34±0.21 | ns |
| LVIDs (mm) | 4.42±0.15 | 4.24±0.33 | 4.16±0.38 | ns |
| RWT | 0.4±0.007 | 0.46±0.04 | 0.46±0.01 | ns |
| LVMI | 3.19±0.28 | 3.51±0.13 | 3.38±0.10 | ns |
| EF% | 76.25±1.88 | 78.75±2.46 | 78.75±3.75 | ns |
| FS% | 47±1.78 | 49.75±2.46 | 49.75±3.90 | ns |
| LADd (mm) | 6.8±0.01 | 7.03±0.34 | 6.94±0.21 | ns |
| LADs (mm) | 4.91±0.12 | 4.89±0.50 | 4.87±0.20 | ns |

**LVIDd** - Left ventricular internal diameter, Diastolic; **LVIDs** - Left ventricular internal diameter, Systolic; **RWT** - Relative wall thickness; **LVMI**- Left ventricular mass index; **FS** -Fractional shortening; **EF** - Ejection fraction; **LADd**- Left atrium diameter, Diastolic; **LADs** - Left atrium diameter, Systolic. n = 4 for both Aldo and Aldo+Vibit-4 groups. p - The p-value of Mann-Whitney test between Aldo and Aldo+Vibit-4 groups**. *** Sham group is from Ref 1 and is shown as a reference for normal values**.**

**References**

1. Klapper-Goldstein H, Murninkas M, Gillis R, Mulla W, Levanon E, Elyagon S, Schuster R, Danan D, Cohen H, Etzion Y (2020) An implantable system for long-term assessment of atrial fibrillation substrate in unanesthetized rats exposed to underlying pathological conditions. Sci Rep 10: 553. DOI 10.1038/s41598-020-57528-3
